# Risk factors for multi-drug resistant *Acinetobacter baumannii *bacteremia in patients with colonization in the intensive care unit

**DOI:** 10.1186/1471-2334-10-228

**Published:** 2010-07-30

**Authors:** Ji Ye Jung, Moo Suk Park, Song Ee Kim, Byung Hoon Park, Ji Young Son, Eun Young Kim, Joo Eun Lim, Sang Kook Lee, Sang Hoon Lee, Kyung Jong Lee, Young Ae Kang, Se Kyu Kim, Joon Chang, Young Sam Kim

**Affiliations:** 1 Division of Pulmonology, Institute of Chest Disease, Department of Internal Medicine, Yonsei University College of Medicine, Seoul, Republic of Korea

## Abstract

**Background:**

Epidemic outbreaks of multi-drug resistant (MDR) *Acinetobacter baumannii *(AB) in intensive care units (ICUs) are increasing. The incidence of MDR AB bacteremia, which develops as a result of colonization, is increasing through widespread dissemination of the pathogen, and further colonization. We sought to determine risk factors for MDR AB bacteremia in patients colonized with MDR AB in the ICU.

**Methods:**

We conducted a retrospective, observational study of 200 patients colonized with MDR AB in the ICU at Severance Hospital, South Korea during the outbreak period between January 2008 and December 2009.

**Results:**

Of the 200 patients colonized with MDR AB, 108 developed MDR AB bacteremia, and 92 did not. APACHE II scores were higher in bacteremic than non-bacteremic patients at the time of ICU admission and colonization (24.0 vs. 21.6; *P *= 0.035, 22.9 vs. 16.8; *P *< 0.001, respectively). There was no difference between the two groups in the duration of time from ICU admission to colonization (7.1 vs. 7.2 days; *P *= 0.923), but the duration of time at risk was shorter in bacteremic patients (12.1 vs. 6.0 days; *P *= 0.016). A recent invasive procedure was a significant risk factor for development of bacteremia (odds ratio = 3.85; 95% CI 1.45-10.24; *P *= 0.007). Multivariate analysis indicated infection and respiratory failure at the time of ICU admission, maintenance of mechanical ventilation, maintenance of endotracheal tube instead of switching to a tracheostomy, recent central venous catheter insertion, bacteremia caused by other microorganism after colonization by MDR AB, and prior antimicrobial therapy, were significant risk factors for MDR AB bacteremia.

**Conclusions:**

Patients in the ICU, colonized with MDR AB, should be considered for minimizing invasive procedures and early removal of the invasive devices to prevent development of MDR AB bacteremia.

## Background

*Acinetobacter baumannii *(AB) is emerging as an important pathogen, especially in intensive care units (ICUs). The increasing development of multiple antimicrobial resistances in this pathogen has severely restricted the therapeutic options available for infected patients, and increased the length of stay in ICUs and mortality [1,2]. Despite intensive efforts, nosocomial acquisition of multi-drug resistant (MDR) AB is still a problem due to the great ability of AB to disseminate from and colonize human and environmental reservoirs [3,4].

Various studies using different methodologies have analyzed risk factors associated with the acquisition of AB. Most of them have addressed factors that influence the risk of infection with MDR AB, comparing to infection with non-MDR AB, or non-AB [3-9]. These factors include prior colonization, which was independently related to the development of MDR AB bacteremia [3,9], and colonization [3]. However, there is limited data on risk factors associated with the development of MDR AB bacteremia from colonization in ICUs.

Recently, major endemic and epidemic outbreaks of MDR AB have developed in critically ill patients throughout the world; aggressive control measures to prevent the transmission and colonization of this pathogen are currently limited. The incidence of MDR AB bacteremia has increased [10,11]; thus, efforts to identify factors that influence the survival of patients with this pathology have been made [2,12,13]. It is known that mortality increases with each hour that appropriate antimicrobial therapy is delayed in patients with septic shock [14]. In several studies, inappropriate, empirical, antimicrobial therapy was independently associated with poor clinical outcome, and early, appropriate, antimicrobial therapy was shown to improve survival in patients with an MDR AB bloodstream infection [2,12,13].

We conducted a retrospective, observational study among patients colonized with MDR AB admitted to our ICU to assess risk factors associated with the development of MDR AB nosocomial bacteremia. Knowledge of these risk factors will allow recommendations for preventive and therapeutic guidelines for the patients colonized with MDR AB.

## Methods

### Study subjects

This was a retrospective, observational study of 200 patients colonized with MDR AB, admitted to the medical ICUs of Severance Hospital, Seoul, South Korea (a university, tertiary, referral hospital with two 15-bed medical ICUs) from January 2008 to December 2009. The outbreak of MDR AB infection has been developed in the ICUs since 2008. The finger print polymerase chain reactions for the isolated AB from the patients' blood and from the environment were randomly performed and the concordance in the types was observed.

During the study period, a total of 903 patients were admitted to the ICUs. Screening cultures were performed in blood, urine, and sputum/endotracheal aspirate for all the patients at the time of ICU admission. During the ICU stay, additional cultures were performed every 4 to 5 days and when infection signs such as systemic inflammatory response syndrome were newly developed, or sustained for more than 3 days after antibiotics change, or when clinical deterioration such as worsening of fever, respiratory condition, and/or radiographic status, requiring mechanical ventilation, requiring aggressive fluid resuscitation or vasopressors were observed.

**Figure 1:**
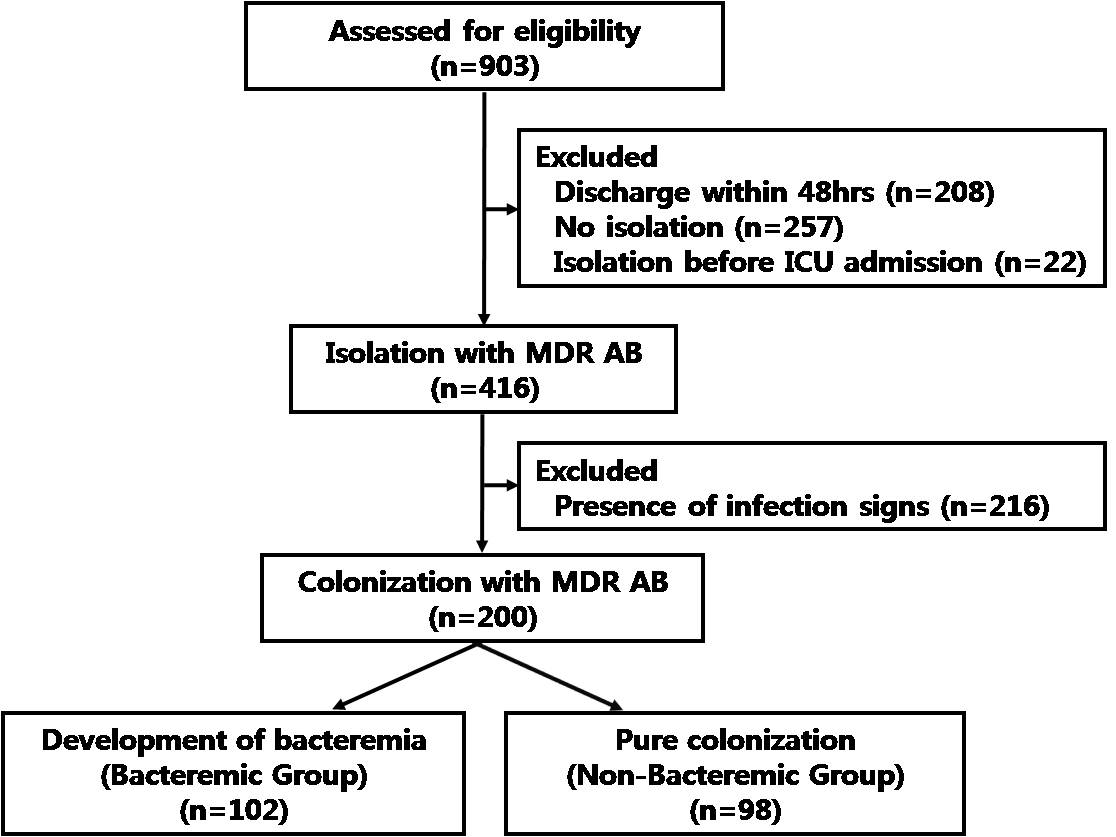
**Summary of the study design**. ICU intensive care unit. MDR multi-drug resistant. AB *Acinetobacter baumannii*.

Of 903 patients, 208 (23.0%) were discharged within 48 h of ICU admission, 22 (2.4%) had been previously colonized by MDR AB before ICU admission, 257 (28.5%) had never been colonized by MDR AB, and 416 (46.0%) were isolated with MDR AB after ICU admission. Of these 416 patients, 216 (23.8%) were excluded because they showed the possibility of infection with MDR AB, according to the surveillance definition of the Centers for Disease Control and Prevention/National Healthcare Safety Network [15]. Twenty-three patients developed AB bacteremia without prior colonization and 6 patients developed non-MDR AB bacteremia. After excluding these patients, the study population consisted of the remaining 200 patients colonized with MDR AB (Figure 1). All of the colonization was diagnosed by endotracheal aspirate culture and colonization of 18 patients by urine culture at the same time.

### Study Design

Baseline characteristics and various risk factors were compared between bacteremic and non-bacteremic patients. The risk factors were as follows: underlying illness, primary ICU admission diagnosis, acquisition types of infection, Acute Physiological Assessment and Chronic Health Evaluation (APACHE II) score, duration of hospitalization, recent invasive procedures, presence of invasive devices, prior antimicrobial therapy, immunosuppression, and bacteremia due to other pathogens before and after colonization by MDR AB. This study protocol was approved by the Ethical Review Committee of Severance Hospital.

### Definitions

Colonization was defined as the presence of microorganisms on skin, on mucous membranes, in open wounds, or in excretions or secretions but are not causing adverse clinical signs or symptoms according to the surveillance definition of the Centers for Disease Control and Prevention/National Healthcare Safety Network [15].

Nosocomial ICU-acquired bacteremia was defined on the basis of the isolation of one or more microorganisms from blood cultures 48 h after admission to the ICU with systemic inflammatory response syndrome (SIRS) [16]. SIRS was defined as the presence of microbes or their toxins in the blood, or two or more of the following conditions: (1) body temperature > 38°C or < 36°C; (2) leukocytosis (> 10,000/mm^3^), leucopenia (< 4,000/mm^3^), or > 10% bands; (3) heart rate > 90 beats/min; or (4) respiratory rate > 24 breaths/min [17]. Only the first isolation was considered.

The duration of exposure to risk was defined as the time from colonization to the date a positive-culture was obtained for MDR AB for bacteremic patients, or the date of discharge from the ICU for non-bacteremic patients.

The microorganism was defined as MDR if it was not susceptible to more than three of the following eight antimicrobial agents: ampicillin/sulbactam, aztreonam, ceftazidime, ciprofloxacin, gentamicin, imipenem, piperacillin, and trimethoprim/sulfamethoxazole [18,19]. Antibiotic sensitivity was determined using the disc diffusion method, according to the Clinical and Laboratory Standards Institute guidelines [20].

The significant coagulase-negative *Staphylococcus *(CNS) bacteremia was defined as isolation of the microorganism from 2 different blood cultures and showing clinical signs of infection at the same time.

Immunosupression was defined as a history of any of the following: corticosteroid therapy for 15 days (at least 10 mg/day of prednisone or an equivalent drug), seropositivity for human immunodeficiency virus, solid organ transplantation, bone marrow transplantation, radiation therapy or chemotherapy for an underlying malignancy during the 6 months prior to hospital admission, and acquired immune deficiency disorder (hypogammaglobulinemia, combined variable immunodeficiency).

Neutropenia was defined as absolute neutrophil count < 1,500 cells/μℓ blood.

Prior utilization of healthcare services included ICU admission or hospitalization within 90 days of the present ICU admission.

Recent invasive procedure was defined as the use of an invasive procedure (arterial catheter, central venous catheter, abdominal drainage catheter, thoracic drainage catheter/tube, nasogastric tube, urinary catheter, continuous renal replacement therapy (CRRT), peritoneal dialysis, tracheostomy, and endotracheal tube) within the 48 h preceding the date of the positive culture for MDR AB for bacteremic patients, or the date of discharge from the ICU for non-bacteremic patients.

The presence of an invasive device was defined as having a device related to the invasive procedure for at least 48 h within the 2 weeks preceding the date of the positive culture for MDR AB for bacteremic patients, or the date of discharge from the ICU for non-bacteremic patients.

Prior antimicrobial therapy was defined as the use of a systemic antimicrobial agent for at least 72 h within the 2 weeks preceding the date of the positive-culture for MDR AB for bacteremic patients, or the date of discharge from the ICU for non-bacteremic patients.

### Statistical analyses

Distribution of continuous variables was tested, and that normally and non-normally distributed variables are presented as mean ± standard deviation and median (interquartile range), respectively. Categorical variables were analyzed using the χ^2 ^test or Fisher's exact test, and continuous variables were analyzed using Student's *t*-test or Mann-Whitney U test. Multivariate analysis was performed using a logistic regression model with interaction impacts to estimate the risk factors of occurrence of MDR AB bacteremia and is presented with an odds ratio (95% confidence intervals, CI). Potential candidate variables were those with *P *< 0.05 in univariate analyses. All tests were two-sided, and a *p-*value < 0.05 was deemed to indicate statistical significance. SPSS 12.0 (SPSS, Chicago, IL, USA) was used for all statistical analyses.

## Results

### Patient characteristics

Baseline characteristics of the study population are shown in Table 1. Of the 200 patients colonized with MDR AB, 108 (54.0%) developed MDR AB bacteremia, and 92 (46.0%) did not. Among 108 episodes of MDR AB bacteremia, carbapenem sensitive AB was observed in 15 (13.9%) patients. There was no difference between the two groups in terms of age or gender. Significantly more patients with bacteremia had malignancies and organ/bone marrow transplants as underlying illnesses, and significantly more patients without bacteremia had central nervous system disorders. The most common reason for ICU admission in both groups was infection. Infection (94.4% vs. 71.7%; *P *< 0.001) and respiratory failure (39.8% vs. 8.7%; *P *< 0.001) were more frequently observed in bacteremic than in non-bacteremic patients. Infection in 56.1% of non-bacteremic patients was community-acquired, and in 45.1% of bacteremic patients was hospital-acquired (*P *= 0.037).

**Table 1:** Demographic Data of Non-Bacteremic and Bacteremic Patients^a^

Parameters	Non-Bacteremic (n = 92)	Bacteremic (n = 108)	*P*-value
Age (yrs)	61.9 ± 15.6	61.6 ± 15.4	0.865
Gender			
Men Women	55 (59.8) 64 (59.3)	37 (40.2) 44 (40.7)	0.940
**Underlying illness**			
Diabetes mellitus	29 (31.5)	32 (29.6)	0.772
Lung disease	19 (20.7)	12 (11.1)	0.063
Central nervous system disorders	28 (30.4)	13 (12.0)	0.001
Renal disease	10 (10.9)	12 (11.1)	0.957
Hypertension	36 (39.1)	39 (36.1)	0.660
Cardiovascular disease	16 (17.4)	13 (12.0)	0.284
Liver disease	5 (6.5)	15 (13.9)	0.09
Rheumatologic disease	0 (0)	4 (3.7)	0.062
Malignancy	18 (19.6)	64 (59.3)	< 0.001
Solid organ malignancy	14 (15.2)	33 (30.6)	0.011
Hematologic malignancy	5 (5.4)	31 (28.7)	< 0.001
HIV infection	0 (0)	2 (1.9)	0.190
Organ or Bone Marrow Transplantation	1 (1.1)	12 (11.1)	< 0.001
**Primary ICU admission diagnosis**			
Infection	66 (71.7)	102 (94.4)	< 0.001
Respiratory failure	8 (8.7)	43 (39.8)	< 0.001
Pulmonary bleeding	2 (2.2)	1 (0.9)	0.595
Gastrointestinal bleeding	3 (3.3)	3 (2.8)	1.000
Liver failure	8 (8.7)	3 (2.8)	0.067
Cardiologic disease	3 (3.3)	2 (1.9)	0.663
Endocrinologic disease	1 (1.1)	0 (0)	0.460
Other	8 (8.7) ^b^	9 (8.3) ^c^	0.927
**Acquisition of Infection **^d^			
Community-acquired	37 (56.1)	37 (36.3)	0.037
Hospital-acquired	19 (28.8)	46 (45.1)	
Healthcare-associated	10 (15.1)	19 (18.6)	

^a ^Data are presented as numbers (percentages) unless otherwise indicated. Plus-minus values are means ± standard deviation

^b ^Mental change (6), post-operative care (2)

^c ^Renal failure (3), brain hemorrhage (2), mental change (2), post-operative care (2)

^d ^Number of patients/total number of patients who were admitted to ICU due to infection (percentages)

HIV human immunodeficiency virus

ICU intensive care unit

### Risk factors related to clinical characteristics

Table 2 shows the univariate analysis of risk factors related to clinical characteristics. Immunosupression, neutropenia, and APACHE II score at ICU admission were significant risk factors. APACHE II score, shock, and acute renal failure at the time of colonization were significantly correlated with the development of MDR AB bacteremia. After colonization, it took about 6 days to develop bacteremia. The duration of time at risk was longer in non-bacteremic patients than in bacteremic patients (12.1 ± 19.3 vs. 6.0 ± 11.1 days; *P *= 0.016). The intubation rate was not different between the groups, but maintenance of mechanical ventilation was a significant risk factor, with an odds ratio of 31.2 (95% CI 14.14-68.83; *P *< 0.001). Furthermore, the longer the mechanical ventilation was maintained, the further the progression of bacteremia (odds ratio = 1.04; 95% CI 1.01-1.08; *P *= 0.018). It took about 7 days to be colonized with MDR AB from the time of ICU admission in both groups.

**Table 2:** Risk factors related to clinical characteristics ^a^

Parameters	Non-Bacteremic (n = 92)	Bacteremic (n = 108)	Odds Ratio	95% CI	*P*-value
Immunosuppressive status ^b^	45 (48.9)	89 (82.4)	4.89	2.57 - 9.29	< 0.001
Neutropenia ^c^	2 (2.2)	25 (23.1)	13.55	3.11 - 59.0	0.001
**Prior history of**					
ICU admission	2 (2.2)	6 (5.6)	2.65	0.52 - 13.45	0.240
Hospitalization	24 (26.1)	41 (38.0)	1.73	0.95 - 3.18	0.075
**APACHE II score at the time of**					
ICU admission (point)	21.6 ± 8.2	24.0 ± 7.9	1.04	1.00 - 1.08	0.035
Colonization (point)	17.0 (12.0 - 20.0)	22.0 (17.0 - 28.0)	1.12	1.07 - 1.17	<0.001
**Duration (days)**					
Of hospitalization	31.0 (20.0 - 65.3)	32.0 (20.0 - 5.0)	0.99	0.99 - 1.00	0.333
Of ICU hospitalization	19.2 ± 21.3	22.8 ± 20.4	1.01	0.99 - 1.02	0.234
From ICU admission to colonization	7.1 ± 7.7	7.2 ± 5.9	1.00	0.96 - 1.01	0.923
Of exposure to risk^d^	6.0 (3.0 - 13.8)	3.0 (1.0 - 6.0)	0.96	0.94 - 0.99	<0.001
**Mechanical ventilator care**	87 (94.6)	105 (97.2)	2.01	0.47 - 8.66	0.348
Maintenance of MV^e^	15 (17.2)	91 (86.7)	31.2	14.14 - 68.83	< 0.001
Duration of MV (day)	9.0 (5.0 - 16.0)	21.5 (12.3 - 47.0)	1.04	1.01 - 1.08	0.001
**Shock at the time of**					
ICU admission	66 (71.7)	80 (74.1)	1.13	0.60 - 2.10	0.711
Colonization	28 (30.4)	65 (60.2)	3.46	1.92 - 6.22	< 0.001
**Acute renal failure at the time of**					
ICU admission	22 (23.9)	31 (28.7)	1.28	0.68 - 2.42	0.445
Colonization	20 (21.7)	41 (38.0)	2.20	1.17 - 4.14	0.014

^a ^Data are presented as numbers (percentages for categorical variables or interquartile range for continuous variables) unless otherwise indicated. Plus-minus values are means ± standard deviation

^b ^Immunosupression was defined as a history of any of the following: corticosteroid therapy for 15 days (at least 10 mg/day of prednisone or an equivalent drug), seropositivity for human immunodeficiency virus, solid organ transplantation, bone marrow transplantation, radiation therapy or chemotherapy for an underlying malignancy during the 6 months prior to hospital admission, and acquired immune deficiency disorder (hypogammaglobulinemia, combined variable immunodeficiency).

^c ^Neutropenia is defined as absolute neutrophil count measured in cells per microliter of blood less than 1,500

^d ^Time from colonization to the date of discharge from the intensive care unit for non-bacteremic patients or to the date a positive-culture was obtained for bacteremic patients

^e ^Number of patients/total number of patients with mechanical ventilation (percentages)

ICU intensive care unit

APACHE Acute Physiological Assessment and Chronic Health Evaluation

MV mechanical ventilation

CI confidential interval

### Risk factors related to recent invasive procedures and presence of invasive devices

Table 3 shows the univariate analysis of risk factors related to recent invasive procedures and the presence of invasive devices. Receipt of any one of the invasive procedures within 48 h of the date of a positive-culture for bacteremic patients or the date of discharge from the ICU for non-bacteremic patients, was a significant risk factor for MDR AB bacteremia (odds ratio = 3.85; 95% CI 1.45-10.24; *P *= 0.007). Patients in whom either an arterial catheter or a central venous catheter was inserted, or patients who received CRRT were at risk of MDR AB bacteremia. Among the inserted invasive devices, central venous catheter, CRRT catheter, and thoracic drainage catheter/tube were significant risk factors. Patients in whom an endotracheal tube was maintained developed MDR AB bacteremia significantly more than those who were switched to a tracheostomy tube.

**Table 3:** Risk factors related to recent invasive procedures and presence of invasive devices^a^

Parameters	Non-Bacteremic (n = 92)	Bacteremic (n = 108)	Odds Ratio	95% CI	*P*-value
**Recent invasive procedure**^b ^	75 (81.5)	102 (94.4)	3.85	1.45 - 10.24	0.007
Arterial catheter	4 (4.3)	15 (13.9)	3.55	1.13 - 11.10	0.030
Central venous catheter	6 (6.5)	29 (26.9)	5.26	2.08 - 13.34	< 0.001
Abdominal drainages	1 (1.1)	5 (4.6)	-	-	-
Thoracic drainage	0 (0)	2 (1.9)	-	-	-
Nasogastric tube	18 (19.6)	11 (10.2)	0.47	0.21 - 1.05	0.064
Urinary catheter	11 (12.0)	17 (15.7)	1.38	0.61 - 3.11	0.443
CRRT	4 (4.3)	27 (25.0)	7.33	2.46 - 21.9	< 0.001
Peritoneal dialysis	2 (2.2)	0 (0)	-	-	-
Tracheostomy	1 (1.1)	3 (2.8)	-	-	-
Other procedures	3 (3.3) ^c^	7 (6.3) ^d^	1.39	0.44 - 4.41	0.574
**Presence of invasive device**^e^	92 (100)	108 (100)	-	-	-
Arterial catheter	84 (91.3)	96 (88.9)	0.76	0.29 - 1.95	0.571
Central venous catheter	82 (89.1)	107 (99.1)	13.05	1.64 - 103.99	0.015
Abdominal drainages	3 (3.3)	5 (4.6)	1.44	0.34 - 6.19	0.624
Thoracic drainage	4 (4.3)	18 (16.7)	4.40	1.43 - 13.52	0.006
Nasogastric tube	90 (97.8)	106 (98.1)	1.18	0.16 - 8.53	0.871
Urinary catheter	91 (98.9)	105 (97.2)	0.39	0.04 - 3.76	0.412
CRRT	7 (7.6)	36 (33.3)	6.07	2.55 - 14.47	< 0.001
Peritoneal dialysis	2 (2.2)	1 (0.9)	0.42	0.04 - 4.71	0.482
Endotracheal tube	46 (55.4)	80 (76.9)	2.72	1.47 - 5.04	0.001
Other devices	2 (2.2)^f^	1 (0.9)^g^	0.42	0.04 - 4.71	0.482

^a ^Data are presented as numbers (percentages) unless otherwise indicated

^b ^Use of an invasive procedure within the 48 h preceding the date of the positive culture for MDR AB for bacteremic patients, or the date of discharge from the ICU for non-bacteremic patients

^c ^Pericardial drainage catheter insertion (1), buttock abscess drainage catheter insertion (1), fiberoptic bronchoscopy (1)

^d ^Fiberoptic bronchoscopy (3), esophagogastroduodenoscopy (1), extracorporeal membrane oxygenation catheter insertion (1), spinal tapping (1), tracheoscopy (1)

^e ^Having a device related to the invasive procedure for at least 48 h within the 2 weeks preceding the date of the positive culture for MDR AB for bacteremic patients, or the date of discharge from the ICU for non-bacteremic patients

^f ^Pericardial drainage catheter (1), buttock abscess drainage catheter (1)

^g ^Extracorporeal membrane oxygenation catheter (1)

CRRT continuous renal replacement therapy

CI confidential interval

### Risk factors related to bacteremia with other microorganisms

Table 4 shows risk factors related to bacteremia caused by other microorganisms before and after colonization by MDR AB. Bacteremia developing in both periods was a significant risk factor for MDR AB bacteremia, with odds ratios of 2.59 (95% CI 1.35-4.99; *P *= 0.004) and 3.06 (95% CI 1.48-6.36; *P *= 0.004), respectively. Before colonization by MDR AB, bacteremia caused by other microorganisms was observed in 37.0% of bacteremic and 18.5% of non-bacteremic patients. CNS (38.0%) was the most common pathogen in bacteremic patients, and fungus (23.5%) in non-bacteremic patients. During the period of time at risk after colonization, bacteremia was observed in 31.5% of bacteremic and 13.0% of non-bacteremic patients. *Enterococcus *species (51.5%) were most commonly isolated from bacteremic patients and CNS (33.3%) from non-bacteremic patients.

**Table 4:** Risk factors related to bacteremia with other microorganisms^a^

Parameters	Non-Bacteremic (n = 92)	Bacteremic (n = 108)	Odds Ratio	95% CI	*P*-value
**Prior other bacteremia before colonization**	17 (18.5)	40 (37.0)	2.59	1.35 - 4.99	0.004
MRSA	1 (5.9)	2 (5.1)	1.72	0.15 - 19.25	0.661
MSSA	2 (11.8)	1 (2.6)	0.42	0.38 - 4.71	0.482
CNS	1 (5.9)	12 (30.8)	12.45	1.59 - 97.14	0.016
*Streptococcus pneumonia*	0 (0)	1 (2.6)	-	-	-
*Streptococcus *other	2 (11.8)	3 (7.7)	1.29	0.21 - 7.87	0.786
*Enterococcus *species	2 (11.8)	9 (23.1)	4.09	0.86 - 19.44	0.076
*Pseudomonas aeruginosa*	1 (5.9)	2 (5.1)	1.72	0.15 - 19.25	0.661
*Escherichia coli*	3 (17.6)	8 (20.5)	2.37	0.61 - 9.22	0.212
*Klebsiella pneumonia*	0 (0)	3 (7.7)	-	-	-
*Enterobacter *species	1 (5.9)	1 (2.6)	0.85	0.05 - 13.79	0.910
*Stenotrophomonas maltophilia*	1 (5.9)	1 (2.6)	1.28	0.59 - 2.77	0.532
*Serratia marcescens*	2 (11.8)	1 (2.6)	0.42	0.04 - 4.71	0.482
Other Gram-positive pathogens	0 (0)	0 (0)	-	-	-
Other Gram-negative pathogens^b^	0 (0)	3 (7.7)	-	-	-
Fungus	4 (23.5)	8 (20.5)	1.76	0.51 - 6.05	0.369
Aanerobes	0 (0)	0 (0)	-	-	-
Polymicrobial bacteremia	1 (5.9)	3 (7.7)	2.60	0.27 - 25.43	0.412
**Prior other bacteremia after colonization**	12 (13.0)	34 (31.5)	3.06	1.48 - 6.36	0.003
MRSA	0 (0)	1 (3.0)	-	-	-
MSSA	2 (16.7)	2 (6.1)	0.85	0.12 - 6.15	0.871
CNS	4 (33.3)	10 (30.3)	2.25	0.68 - 7.41	0.185
*Streptococcus pneumonia*	0 (0)	0 (0)	-	-	-
*Streptococcus *other	0 (0)	0 (0)	-	-	-
*Enterococcus *species	1 (8.3)	17 (51.5)	18.2	2.38 - 139.22	0.005
*Pseudomonas aeruginosa*	0 (0)	5 (12.1)	-	-	-
*Escherichia coli*	0 (0)	1 (3.0)	-	-	-
*Klebsiella pneumonia*	1 (8.3)	1 (3.0)	0.85	0.05 - 13.79	0.909
*Enterobacter *species	0 (0)	1 (3.0)	-	-	-
*Stenotrophomonas maltophilia*	1 (8.3)	1 (3.0)	0.85	0.05 - 13.79	0.909
*Serratia marcescens*	0 (0)	1 (3.0)	-	-	-
Other Gram-positive pathogens	0 (0)	0 (0)	-	-	-
Other Gram-negative pathogens^c^	1 (8.3)	0 (0)	-	-	-
Fungus	1 (8.3)	7 (21.2)	6.31	0.76 - 52.25	0.088
Aanerobes^d^	1 (8.3)	0 (0)	-	-	-
Polymicrobial bacteremia	0 (0)	6 (17.6)	-	-	-

^a ^Data are presented as numbers (percentages) unless otherwise indicated

^b ^Chryseobacterium indologenes, Morganella morganii, Citrobadter freundii

^c ^Chryseobacterium meningosepticum

^d ^Norcardia farcinica

MRSA methicillin-resistant *Staphylococcus aureus*

MSSA methicillin-sensitive *Staphylococcus aureus*

CNS coagulase negative *Staphylococcus*

CI confidential interval

### Risk factors related to prior antimicrobial therapy

Table 5 shows risk factors related to prior antimicrobial therapy. The average number of prior antimicrobials used was 3.8 in bacteremic patients and 2.7 in non-bacteremic patients (*P *< 0.001). Quinolones, carbapenems, glycopeptides, and aminoglycosides were more frequently used in bacteremic than in non-bacteremic patients.

**Table 5:** Risk factors related to prior antimicrobial therapy^a^

	Non-Bacteremic (n = 92)	Bacteremic (n = 108)	Odds Ratio	95% CI	*P*-value	Non-Bacteremic (days)	Bacteremic (days)	*P*-value
Number (percentage) of prior exposure antibiotics/duration of exposure, days	2.7 ± 1.1	3.8 ± 1.4	2.09	1.60 - 2.74	< 0.001	19.2 ± 21.3	13.2 ± 12.9	0.005
Aminopenicillin	2 (2.2)	3 (2.8)	1.29	0.21 - 7.87	0.79	9.5 ± 2.1	6.7 ± 6.4	0.601
Cephalosporin	34 (37.0)	34 (31.5)	0.78	0.44 - 1.41	0.416	11.2 ± 9.1	7.9 ± 6.8	0.09
Quinolone	37 (40.2)	50 (55.6)	1.86	1.06 - 3.27	0.031	11.6 ± 6.2	7.8 ± 3.4	0.002
Antipseudomonal penicillin	34 (37.0)	44 (40.7)	1.17	0.66 - 2.08	0.585	10.6 ± 5.1	6.3 ± 2.9	< 0.001
Carbapenem	45 (48.9)	83 (76.9)	3.47	1.89 - 6.36	< 0.001	14.1 ± 14.9	9.2 ± 6.0	0.003
Glycopeptide	52 (56.5)	85 (78.7)	2.84	1.53 - 5.28	0.001	12.6 ± 7.6	9.3 ± 7.8	0.018
Aminoglycoside	10 (10.9)	30 (27.8)	3.15	1.45 - 6.88	0.004	8 ± 4.1	5.6 ± 3.8	0.099
Anti-anaerobe	24 (26.1)	38 (35.2)	1.54	0.84 - 2.83	0.167	9.0 ± 4.6	6.3 ± 4.1	0.017
Macrolide	2 (2.2)	5 (4.6)	2.18	0.41 - 11.54	0.357	5.5 ± 3.5	4.4 ± 2.6	0.660

^a ^Data are presented as numbers (percentages) unless otherwise indicated. Plus-minus values are means ± standard deviation

CI confidential interval

SD standard deviation

### Multivariate analysis of risk factors for multi-drug resistant Acinetobacter baumannii bacteremia

Table 6 shows the results of the multivariate analysis of risk factors for MDR AB bacteremia. Five independent risk factors associated with MDR AB bacteremia were identified: ICU admission due to infection (odds ratio = 12.04; 95% CI 1.79-80.74; *P *= 0.01), ICU admission due to respiratory failure (odds ratio = 9.35; 95% CI 2.16-40.45; *P *= 0.003), recent central venous catheter insertion (odds ratio = 10.40; 95% CI 1.98-54.75; *P *= 0.006), bacteremia caused by other microorganisms after MDR AB colonization (odds ratio = 4.89; 95% CI 1.37-17.47; *P *= 0.014), and number of prior antimicrobials used (odds ratio = 2.12; 95% CI 1.33-3.39; *P *= 0.002). The interaction was observed only between the variables of maintenance of mechanical ventilation and maintenance of an endotracheal tube instead of switching to tracheostomy, but combined two factors was a significant risk factor for MDR AB bacteremia (odds ratio = 16.64; 95% CI 1.64-168.83; *P *= 0.017).

**Table 6:** Multivariate analysis of risk factors for multi-drug resistant *Acinetobacter baumannii *bacteremia

Risk Factors	Odds ratio	95% CI	*P*-value
Reason for ICU admission			
Infection	12.04	1.79 - 80.74	0.010
Respiratory failure	9.35	2.16 - 40.45	0.003
Maintenance of mechanical venitlation	10.36	2.06 - 52.25	0.005
Maintenance of endotracheal tube	2.19	0.45 - 10.57	0.331
Recent centeral venous catheter insertion	10.40	1.98 - 54.75	0.006
Bacteremia of other microorganisms after colonization	4.89	1.37 - 17.47	0.014
Number of prior antibiotics used	2.12	1.33 - 3.39	0.002
Maintenance of mechanical ventilation × maintenance of endotracheal tube ^a^	16.64	1.64 - 168.83	0.017

^a ^Interaction impacts of maintenance of mechanical ventilation and maintenance of endotracheal tube instead of a switch to tracheostomy

ICU intensive care unit

CI confidential interval

## Discussion

This was a retrospective, observational study to determine risk factors for MDR AB nosocomial bacteremia in patients colonized with MDR AB after ICU admission. Our study showed that the presence of infection and respiratory failure at the time of ICU admission, recent central venous catheter insertion, bacteremia caused by other microorganisms after colonization by MDR AB, and prior antimicrobial therapy, were the independent risk factors for MDR AB bacteremia. Moreover, combined factors of maintenance on a mechanical ventilator and maintenance of an endotracheal tube instead of switching to a tracheostomy, increased the risk of MDR AB bacteremia.

Risk factors for AB bacteremia in the ICU have previously been demonstrated with case-control and cohort methodologies. Multivariate analysis identified male gender, APACHE II score, length of stay in the ICU, mechanical ventilation, prior infection, antimicrobial therapy, prior colonization, and colonization pressure as independent risk factors for AB bacteremia [4,6,7,9]. Recently, epidemic outbreaks of MDR AB have occurred in different areas of hospitals, primarily the ICU, with widespread dissemination and colonization [10,19]. However, knowledge of which patients previously colonized with MDR AB would develop bacteremia in the ICU has been limited. Awareness of the risk factors would help predict bacteremia in colonized patients and allow administration of appropriate antibiotics before culture results are reported. Delay in appropriate antimicrobial therapy has an adverse influence on the clinical outcome of patients with AB bacteremia [12,13,18].

Consistent with other studies [3,4], we found the presence of infection at the time of ICU admission, and bacteremia caused by other microorganisms after MDR AB colonization, independently increased the risk of MDR AB bacteremia 12-and 5-fold, respectively. Infection accompanied by bacteremia caused by other microorganisms reflects underlying disease severity or a requirement for critical care, including invasive interventions. These risk factors are also related to the number of previous antimicrobials used which increased the risk of MDR AB bacteremia by 2-fold in the present study. Treatment with a greater number of broad-spectrum antibiotics is a surrogate marker of illness severity, and could ablate a patient's pre-existing microflora. Duration of exposure to previous antimicrobials was significantly shorter in bacteremic than in non-bacteremic patients, but this result was due to shorter duration of risk exposure. Therefore, the number of previous antimicrobials used at least 72 h within 2 weeks were analyzed in this study.

Our data showed combined factors of maintenance of mechanical ventilation and maintenance of an endotracheal intubation tube without a switch to tracheostomy, increased the risk of bacteremia by almost 17-fold. Mechanical ventilation is the main source of transmission of AB, and AB colonization in the respiratory tract. Tracheobronchitis and pneumonia are known to increase the risk of secondary bacteremia caused by AB in other studies [4-6, 21-23]. In a univariate analysis of risk factors, it was reported that tracheostomy was a significant risk factor for AB colonization [8]. In the present study, it was not identified as a significant risk factor, because only a small number of recent tracheostomies was included.

Recent central venous catheter insertion increased the risk of bacteremia by almost 10-fold. Central venous catheter insertion has previously been reported as a risk factor for AB bacteremia on the basis of univariate analyses [2,6,24]. In this study, using multivariate analysis, it was a significant independent risk factor. The difference is a result of the different types of patients included in the study populations; previous studies compared patients with and without infection of MDR AB. All patients in this study were colonized by MDR AB; thus, the risk of development of bacteremia due to invasive procedures was significant. As with many other invasive procedures, central venous catheter insertion is a major portal of entry as a source of infection [6]. Arterial catheter insertion was a significant risk factor on the basis of a univariate, but not a multivariate, analysis. Although both procedures are performed via a vascular access, the sizes of the puncture needle and guide sheath/wire differ. The chances of developing blood stream infections depend on the length of the devices used and the duration of the procedures.

Previous reports were unclear as to whether a longer stay in the ICU would increase the risk for bacteremia [3,7,8,25-27]. Many other possible risk factors for nosocomial MDR AB bacteremia in the ICU are related to length of hospitalization. To control for the duration of time at risk, we included the time from colonization to the date of a positive-culture for MDR AB for bacteremic patients or to the date of discharge from the ICU for non-bacteremic patients in the final multivariate analysis. The results showed that the duration of exposure to risk was not an independently significant risk factor; in fact, univariate analysis showed it was actually shorter in bacteremic than in non-bacteremic patients. As all the patients in this study were already colonized with MDR AB, the length of stay in the ICU did not affect the development of MDR AB bacteremia; even with a short duration of stay, patients requiring acute care and those maintaining mechanical ventilation and receiving invasive procedures developed bacteremia more frequently.

AB infection or colonization is associated with increased mortality, morbidity, and a prolonged length of stay in the hospital, leading to excessive medical costs [19,25]. Reducing intrinsic contamination and colonization of medical equipment or devices used for monitoring and therapy of patients, and decreasing contamination through airborne or direct contact with patients must be the primary measure used to control the infection of MDR AB in the ICU. Furthermore, attention to various guidelines for the use of care bundles in critical care, such as ventilator bundles, central line bundles, and severe sepsis bundles is important for the prevention of bacteremia in clinical practice, especially for patients colonized with MDR AB. Moreover, efforts to remove invasive devices and equipment such as endotracheal tube or central venous catheter as soon as possible are needed to prevent development of MDR AB bacteremia among the colonized patients [2,12,13].

There are several limitations to the present study. First, this study was processed in a retrospective observational manner; thus, methodological deficiencies in assessing risk factors for MDR AB acquisition may lead to biased estimates or erroneous associations. However, number of patients in this study is larger than previous studies [3,6,21], so potential for bias is expected to be minimized. Second, since active surveillance culture for the presence of AB was not routinely and uniformly performed during ICU stay, not all the patients with colonization may have been included in this study and there may be inaccuracy in the timing of colonization. Third, because outbreak of MDR AB was developed during the study period, this result may not represent overall status of MDR AB bacteremia of the patients with prior colonization in general ward or other intensive care units. Lastly, although we followed the definition of the Centers for Disease Control and Prevention/National Healthcare Safety Network to select the patients colonized by MDR AB, some of the patients included in this study might have been infected with MDR AB, while some of those that were excluded might have been colonized by MDR AB.

## Conclusions

In conclusion, this study demonstrated the several risk factors for MDR AB nosocomial bacteremia in patients with MDR AB colonization in the ICU. Independent risk factors were the presence of infection and respiratory failure at the time of ICU admission, maintenance of mechanical ventilation, maintenance of an endotracheal tube instead of a switch to a tracheostomy, recent central venous catheter insertion, bacteremia caused by other microorganism after colonization with MDR AB, and prior antimicrobial therapy. Patients in the ICU, colonized with MDR AB, should be considered for minimizing invasive procedures and early removal of the invasive devices or equipments to prevent development of bacteremia [2].

## Abbreviations

MDR: multi-drug resistant; AB: *Acinetobacter baumannii*; ICU: intensive care unit; APACHE: Acute Physiological Assessment and Chronic Health Evaluation; SIRS: systemic inflammatory response syndrome; CRRT: continuous renal replacement therapy; SD: standard deviation; CI: confidence interval; CNS: coagulase negative *Staphylococcus*; HIV: human immunodeficiency virus; MV: mechanical ventilation; MRSA: methicillin-resistant *Staphylococcus aureus; *MSSA: methicillin-sensitive *Staphylococcus aureus*

## Competing interests

The authors declare that they have no competing interests.

## Authors' contributions

JJ carried out screening and acquisition of data, statistical analysis and participated in the writing of the manuscript. SEK, SKL and SHL carried out screening and acquisition of data. BP, JS, EK participated in the acquisition of data and statistical analysis. JL, KL participated in the interpretation of data. YK participated in the study design and the analysis and interpretation of data. SKK and JC participated in the study design, analysis and interpretation of data and critical revision of the manuscript for important intellectual content. MP and YK participated in the study design, analysis and interpretation of data and the writing of the manuscript. All authors read and approved the final manuscript.

## Pre-publication history

The pre-publication history for this paper can be accessed here:

http://www.biomedcentral.com/1471-2334/10/228/prepub
